# Rod Rotation with Outrigger Is Substantial for Correcting Apical Hypokyphosis in Patients with Adolescent Idiopathic Scoliosis: Novel Outrigger Device for Concave Rod Rotation

**DOI:** 10.3390/jcm12216780

**Published:** 2023-10-26

**Authors:** Shoji Seki, Hiroto Makino, Yasuhito Yahara, Katsuhiko Kamei, Hayato Futakawa, Taketoshi Yasuda, Kayo Suzuki, Masato Nakano, Yoshiharu Kawaguchi

**Affiliations:** 1 Department of Orthopaedic Surgery, Faculty of Medicine, University of Toyama, Toyama 930-0194, Japan; hiroto@med.u-toyama.ac.jp (H.M.); kame1234@med.u-toyama.ac.jp (K.K.); hayato83@med.u-toyama.ac.jp (H.F.); yasuda@med.u-toyama.ac.jp (T.Y.); suzukayo@med.u-toyama.ac.jp (K.S.); zenji@med.u-toyama.ac.jp (Y.K.); 2 WPI-Immunology Frontier Research Center, Osaka University, Suita, Osaka 565-0871, Japan; yyahara@icb.med.osaka-u.ac.jp; 3 Department of Orthopaedic Surgery, Takaoka City Hospital, Toyama 933-8550, Japan; mnakano-tym@umin.ac.jp

**Keywords:** adolescent idiopathic scoliosis, posterior spinal fusion, rod rotation, differential rod contouring, outrigger, thoracic kyphosis

## Abstract

The apical hypokyphosis of scoliotic patients is thought to lead to decreased lung capacity and cause shortness of breath. Additionally, concave rod curve reduction is a problem in the correction of apical hypokyphosis in posterior spinal fusion surgery in adolescent idiopathic scoliosis (AIS). We investigated the contributions of rod rotation (RR) with an outrigger device, followed by differential rod contouring (DRC) with the outrigger attached to the concave rod, designed to prevent concave rod curve-flattening. We analyzed and compared the results of segmental pedicle screw fixation without the outrigger in 41 AIS patients with thoracic curves (Lenke type I, 25; type II, 16) to those corrected using the outrigger in 36 patients (Lenke type I, 24; type II,12). The changes in the Cobb angle, apical kyphosis of five vertebrae, thoracic kyphosis (TK, T4–12), correction rate, correction angle of apical vertebral rotation, spinal penetration index (SPi), and rib hump index (RHi) before and after surgery were measured, and the contribution of the outrigger was analyzed. The mean scoliosis correction rates without and with the outrigger were 72.1° and 75.6°, respectively (*p* = 0.03). Kyphosis of the five apical vertebrae and TK were significantly greater in the surgery with the outrigger (*p* = 0.002). Significantly greater improvements in SPi and RHi were also noted in the surgery with the outrigger (*p* < 0.05). The use of concave RR and convex DRC with the outrigger appear to be advantageous for correcting apical hypokyphosis, followed by the subsequent formation of TK. As a result, breathing problems are less likely to occur during daily life because of improvements in SPi and RHi.

## 1. Introduction

**Figure 1:**
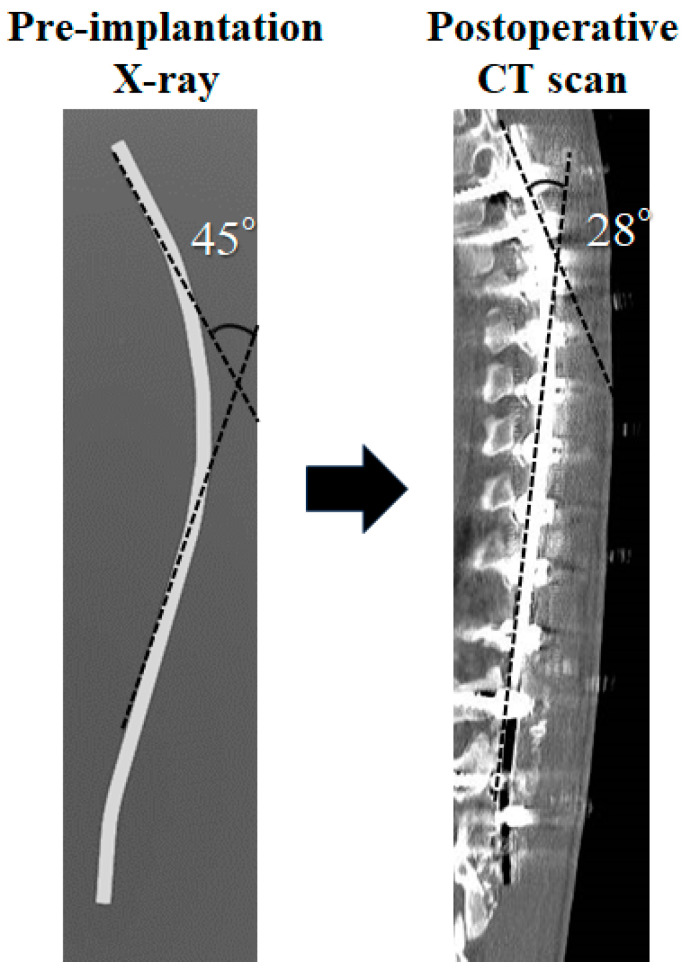
This figure shows concave rod deformation. The concave rod was 45° before surgery. CT scan showed deformations of 28° after surgery. The convex rod was not deformed.

Adolescent idiopathic scoliosis (AIS) of the thoracic spine causes a three-dimensional deformity of the spine and ribcage. The treatment goal should be a well-balanced spine in all planes. The concave rod rotation (RR) technique has been developed in recent decades using Cotrel–Dubousset (CD) instrumentation [1] and the segmental pedicle screw fixation technique with RR, followed by direct vertebral rotation (DVR) [2,3]. Cidambi et al. [4] reported that rod contouring was significantly different between the concave and convex sides after surgery, with decreased curvature in the concave rod. They recommended the compensatory over-contouring of the concave rod for spinal correction in patients with AIS [4]. Differential rod contouring (DRC) is useful in improving vertebral rotation for posterior spinal fusion and correction in patients with AIS. The use of DRC with CoCr rods additionally improved apical rotation by almost 6° of apical rotation for thoracic scoliosis [5,6,7]. Overbending of the concave rod contour was significantly flattened after RR [8,9]. As shown in Figure 1, the concave rod measured 45° pre-implantation, while a postoperative X-ray and CT scan measured 28°. Convex rod deformation was not seen between pre- and postoperative images. CoCr rods have been reported to exhibit considerable stiffness compared to other materials such as titanium alloys and ultra-stainless steel, but they were still deformed after surgery [10,11]. Considering these facts, we considered that the 5.5 mm CoCr rod alone might not be able to sufficiently maintain the corrective force of the scoliotic spine through concave RR. Therefore, it was considered possible that sufficient rotation correction and kyphosis formation could not be achieved because the concave rod was deformed. For the above reasons, we have developed an outrigger device that prevents rod deformation during concave RR.

Although there have been several reports of some attempts to correct the hypokyphosis of the thoracic curvature in patients with AIS [9,12], it seems that there are still not enough reports regarding the correction of apical hypokyphosis. Monazzam et al. [13] reported that 222 out of 280 patients remained hypokyphotic after surgery. The loss of thoracic kyphosis (TK) after surgery in patients with AIS is implicated in the loss of lumbar lordosis [14], resulting in a high incidence of proximal junctional kyphosis [15] and distal junctional kyphosis [16]. The postoperative loss of lumbar lordosis is also associated with lower back pain [17]. Correcting hypokyphosis in patients with AIS increases lung volume and improves pulmonary function [18]. If an over-bent concave rod was flattened during concave RR as mention above, thoracic hypokyphosis was not likely to be corrected. Considering these facts suggests that correcting hypokyphosis and obtaining substantial TK is a necessary to lead a healthy daily life in patients with AIS after surgery.

To prevent concave rod deformation during RR, we developed an outrigger device that can be attached to the concave rod, which temporarily increases its stiffness. Although this device is easy to use, it is still unclear how much it can correct apical hypokyphosis, general scoliotic curvature, and lung volume. We investigated the effect of the outrigger device with concave RR and a convex DRC on the correction of thoracic apical hypokyphosis, along with its attendant decreased lung volume and scoliotic curvature, in patients with AIS.

## 2. Materials and Methods

### 2.1. Subjects

A total of 77 patients were enrolled in this study. Forty-one patients with AIS (39 females, 2 males; mean patient age 14.1 years) underwent concave RR and convex DRC without an outrigger device, followed by DVR using uniplanar screws, at our institution. According to the classification of Lenke et al. [19], Lenke Type 1 and 2 patients were numbered 25 and 16, respectively. Next, 36 consecutive patients with Lenke Type 1 and 2 (32 females, 4 males; mean patient age 14.0 years) underwent surgery from 2017 to 2020 using concave RR and convex DRC with the outrigger device. Mean follow-up periods in the surgery in the groups without and with the outrigger were 6.9 and 3.1 years, respectively. All surgeries were performed by one senior spine surgeon specializing in scoliosis (S.S.). The inclusion criteria were patients with AIS in the age range of 11–20 years with Lenke classification type 1 or 2. The exclusion criteria were patients with severe thoracic deformity due to pectus excavatum, cardiac surgery, severe mental disorders, or neurological disorders such as syringomyelia, and patients undergoing hormonal therapy. The patients were evaluated by X-rays and CT for screw insertion and thoracic deformation before and after surgery. The X-ray data were analyzed before and more than 2 years after surgery. Prior approval of the study was obtained from the Ethical Review Board of University of Toyama (No. 28-108). Informed consent to participate in this study and consent to instrumentation were obtained before surgery.

### 2.2. Surgical Technique

#### 2.2.1. Technique without Outrigger

As described previously [5,6,7], we first performed segmental uniplanar pedicle screw fixation with concave RR, followed by DRC and segmental DVR, in the 41 patients.

#### 2.2.2. Technique with Outrigger

**Figure 2:**
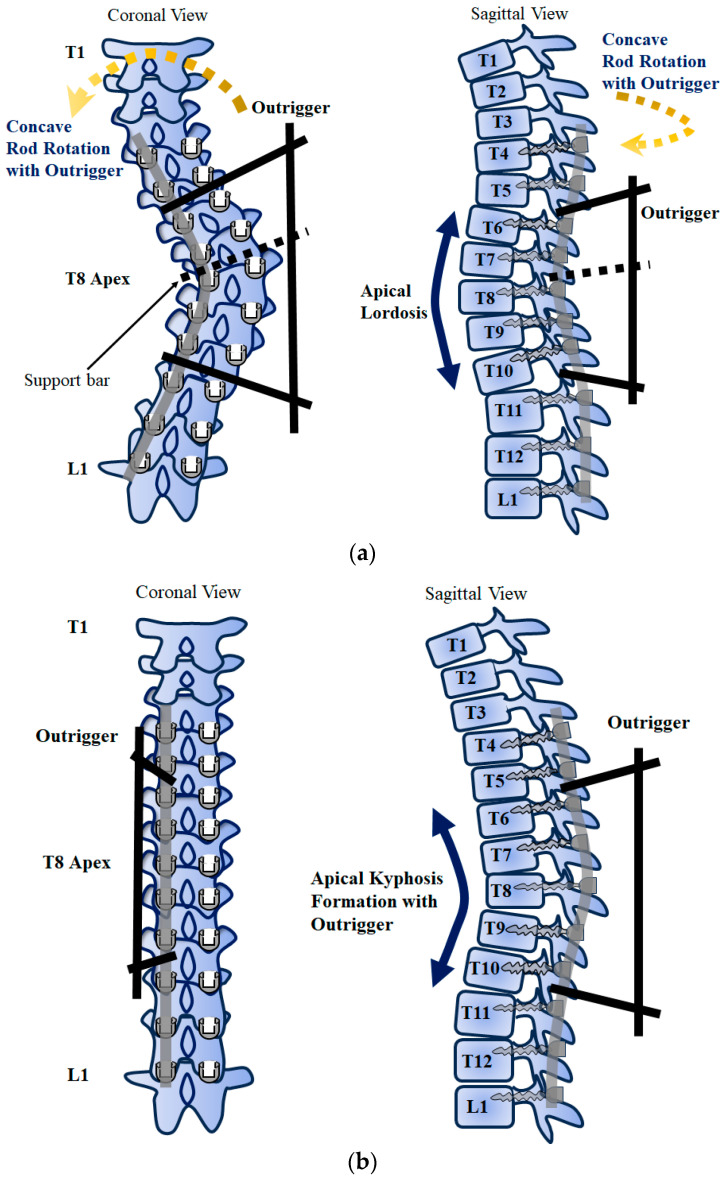
This figure shows the surgical technique with the outrigger device. The outrigger device was attached to the periapical region of the concave rod ((**a**) Schema of scoliosis correction method using outriggers), and then concave RR was performed to correct apical hypokyphosis and subsequently form apical kyphosis ((**b**) Schema of apical kyphosis formation after concave RR). The concave rod sandwiched by the outrigger devices was reinforced to prevent deformation during concave RR. After concave RR with an outrigger device was used, the setscrews sandwiched by the outrigger were tightened, and the support bar was removed. Then, DRC using a convex rod was performed while the outrigger device was attached to the concave rod.

The surgical technique with the outrigger device (shown in Figure 2) was performed in 36 patients in the outrigger group. A posterior, midline, straight incision was made from the uppermost to the lowest vertebra designated for instrumented fusion. After exposing the spine, segmental uniplanar screws were inserted by the free-hand technique, the facet joints of the fusion levels and their articular cartilages were removed, and then Ponte osteotomy [20] was performed on the periapical intervertebral spaces. Next, concave RR with an outrigger device was used (Figure 2a). The outrigger device was attached to the periapical vertebrae so as to sandwich the caudal and cranial neutral vertebrae in a Lenke-type 1,2 AL curve [21]. For the Lenke 1, 2 AR curve [21], the outrigger device was attached to 4 or 5 periapical vertebrae. After the use of concave RR with an outrigger device, the setscrews sandwiched by the outrigger were tightened, and the support bar was removed (Figure 2b). Then, convex DRC was performed while the outrigger device was attached to the concave rod. Both concave and convex rods were 5.5 mm CoCr. The concave rod was overbent by approximately 10° compared to the convex rod. The most important aspect of this surgical method is that the outrigger and the concave rod should be firmly fixed in parallel when performing concave RR using the outrigger. A support bar was used in the middle of the outrigger to ensure smooth parallel rotation (Figure 2a). Next, we performed DVR on each vertebra, from the most caudal neutral vertebra to the lowest instrumented vertebra, for lumbar curve correction. After correction, total spinal balance was checked by fluoroscopy. All patients were evaluated using spinal cord monitoring with motor-evoked and somatosensory-evoked potentials during surgery.

### 2.3. Radiographical and CT Assessment

All patients were evaluated by X-ray and CT scanning before and after surgery. CT scans were used to plan screw insertion and check rib cage deformities and any other spinal anomalies. We used the Synapse Vincent image analyzer (Fujifilm, Tokyo, Japan) to ensure the accuracy of angle measurement in the radiographic image and CT slice. We measured radiographic parameters, such as Cobb angle, TK, lumbar lordosis (LL), apical vertebral translation (AVT), apical vertebral rotation (AVR), and correction rate. Next, we measured pre- and postoperative rib hump index (RHi) and spinal penetration index (SPi) to assess thoracic prominence and lung volume changes from pre- and postoperative CTs based on the method reported by Aaro et al. [22] and Dubousset et al. [23], respectively. After the radiographic parameters were measured, we looked for any association between concave RR with and without the outrigger device.

### 2.4. Statistical Analysis

Data are presented as the mean ± standard deviation. Mann–Whitney’s *U*-test and unpaired *t*-tests were used for the statistical analysis of the differences between concave RR with and without the outrigger device. The RHi and SPi values were also analyzed by Mann–Whitney’s *U*-test. Commercial software (JMP^®^ version 9, SAS Institute Inc., Cary, NC, USA) was used for the analysis, with *p* < 0.05 considered statistically significant. A power analysis determined that a minimum sample size of 31 in each group would require 80% power to detect a difference [24].

### 2.5. Inter-Rater and Intra-Rater Reliability

We investigated the inter-rater and intra-rater reliability of the measurements of both X-ray and CT images by calculating the Fleiss’ Kappa coefficient using a dedicated program (MATLAB Mathworks, Paris, France) [5,6]. Kappa values of 0.00–0.20 were interpreted as slight agreement, 0.21–0.40 were interpreted as fair agreement, 0.41–0.60 were interpreted as moderate agreement, 0.61–0.80 were interpreted as substantial agreement, and 0.81–1.00 were interpreted as almost perfect agreement [25,26]. We also determined inter-rater and intra-rater percentage agreement [27].

## 3. Results

### 3.1. Basic Characteristics of Both Groups

The groups’ preoperative radiological and clinical parameters showed no significant differences in the concave RR and convex DRC (Table 1). The preoperative Cobb angles of the main thoracic (MT) curve in the RR without and with the outrigger were 52.2° and 52.7°, respectively (Table 1).

### 3.2. Surgical Outcomes of Outrigger Device

The surgical outcomes of the RR without and with the outrigger are shown in Table 2. The TK of the RR in the outrigger group was significantly greater than that of the RR in the no outrigger group (*p* = 0.003). The correction rate was also significantly higher in the RR of the outrigger group than that in the RR in the no outrigger group (*p* = 0.02). No significant differences between the groups were observed in the corrected angles of AVR or in the correction rates of thoracic AVT (TAVT). The estimated blood loss during surgery and operation time were significantly reduced for surgery in the outrigger group compared to surgery in the without outrigger group (*p* < 0.05). In the RR with outrigger group, there were no complications such as screw backout, subcutaneous screw protrusion, or neurological deterioration. One patient had signal changes during spinal cord monitoring, but no neurological deterioration was seen in this patient.

### 3.3. Analysis of Changing in TK and Apical Vertebral Kyphosis

**Figure 3:**
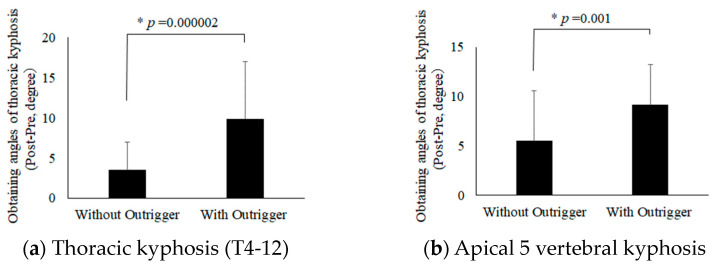
Changes in TK and apical 5 vertebral kyphosis before and after surgery: (**a**) an analysis of differences in the angles of TK with and without the outrigger device; (**b**) an analysis of differences in angles of the kyphosis of the five apical vertebrae with and without the outrigger device.

To investigate the effect of the outrigger on TK, the changes in TK and apical five vertebral kyphosis measured by X-ray before and after surgery were compared between the groups with and without outriggers (Figure 3). In measurements of the postoperative increases in TK, the outrigger group TK increased by 9.9° upon postoperative X-ray (a), while the concave RR in the group the outrigger group increased by only 3.6° (*p* = 0.000002). Similarly in the five apical vertebrae, kyphosis was significantly better preserved in the outrigger group (*p* = 0.001) (b).

### 3.4. An analysis of RHi and SPi with and without the Outrigger

**Figure 4:**
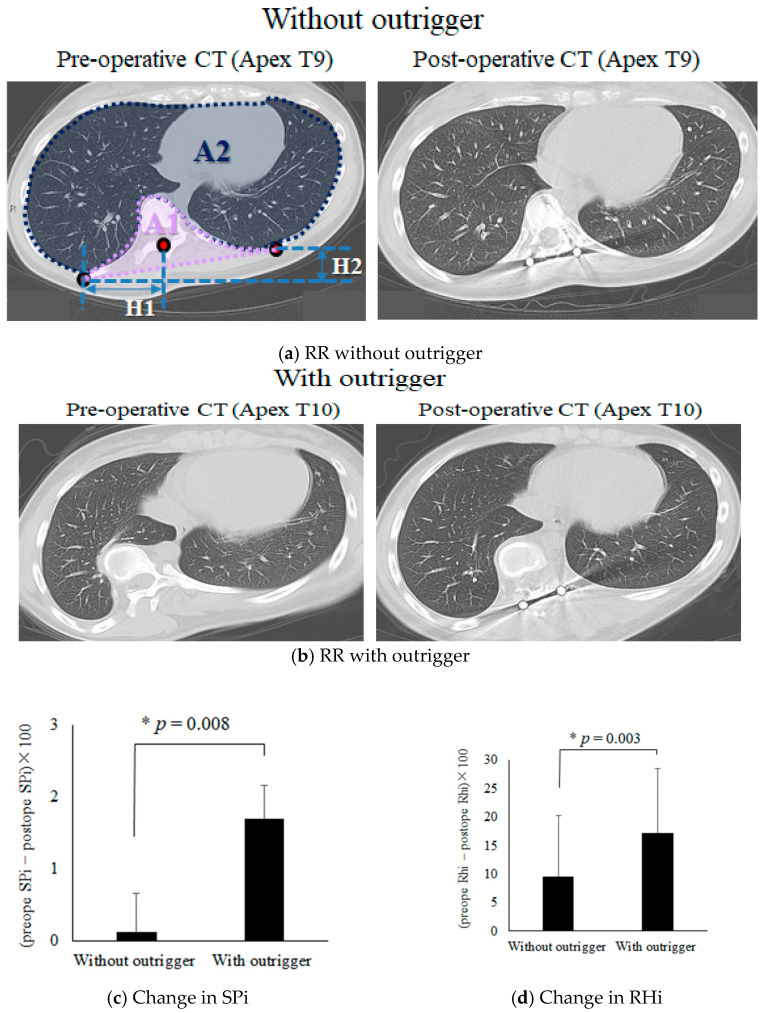
SPi and RHi analyses of RR with and without the outrigger device. SPi was calculated as A1/A2. RHi was calculated as H1/H2. Axial pre- and postoperative CT scans without (**a**) and with (**b**) the outrigger. The lung volume increased after surgery in (**b**). There were significant increases of in SPi (**c**) and RHi (**d**) with the outrigger group compared to those without the outrigger.

We have shown that the use of concave RR followed by convex DRC with the outrigger results in a more normal TK in patients with AIS. In addition, we investigated whether lung volume increased and rib hump decreased using CT scans. The curvature apex of pre- and postoperative axial CT images is shown in Figure 4. (a) The lung volume in the axial plane does not appear to significantly change before and after surgery in the RR without the outrigger. (b) The lung volume, especially on the right side, is clearly increased after concave RR with the outrigger (Figure 4b). (c) The mean change in SPi ([Preoperative SPi − Postoperative SPi] × 100) was 0.1 and +2.0 in the RR without and with the outrigger, respectively. (d) The use of RHi ([Preoperative RHi − Postoperative RHi] × 100), comparing the outrigger and non-outrigger groups, revealed significant increases in SPi (c) and RHi (d) in the outrigger group (*p* < 0.05), suggesting that the increased TK with the outrigger contributed to the increased lung volume and a decreased rib hump.

### 3.5. Inter-Rater and Intra-Rater Reliability

The averaged Fleiss’ Kappa coefficient of inter-rater reliability for the measurement of the intraoperative X-ray and CT images was 0.89 ± 0.02 and 0.81 ± 0.08 for the three observers, respectively. The intra-rater reliability for the measurement of the intraoperative X-ray and CT was 90.1% (95% CI 85.5–95.5) and 91.2% (95% CI 85.5–95.0), respectively.

### 3.6. Representative Cases in RR with Outrigger Device

**Figure 5:**
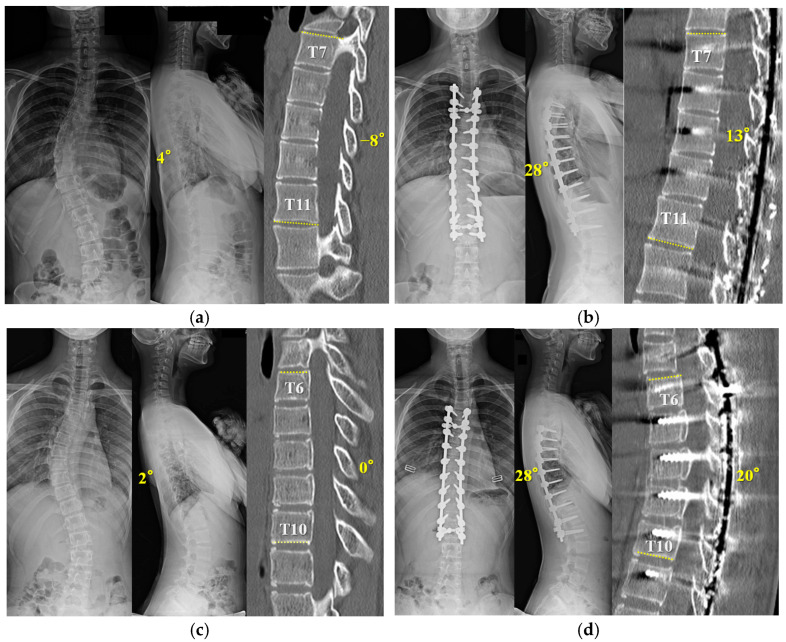
The X-ray and CT show a fifteen-year-old girl with Lenke 1A- scoliosis (**a**,**b**), and a thirteen-year-old girl with Lenke 1B- (**c**,**d**). (**a**) Preoperative X-ray shows a Cobb angle of 48° and 4° of TK. Apical five vertebral kyphosis is −8 degrees. (**b**) Postoperative X-ray shows a Cobb angle of 5°and 28° of TK after posterior fusion and correction using the outrigger. Apical five vertebral kyphosis is improved by 13 degrees. (**c**) Preoperative X-ray shows a Cobb angle of 51° and 2° of TK. Apical five vertebral kyphosis is 0 degrees. (**d**) Postoperative X-ray shows a Cobb angle of 5°and 28° of TK after posterior fusion and correction using the outrigger. Apical five vertebral kyphosis is improved by 20 degrees.

A fifteen-year-old girl with Lenke 1A- was used as the representative case (Figure 5a,b). (a) A preoperative X-ray showed thoracic hypokyphosis of 4°. Apical five vertebral kyphosis was −8 degrees. (b) She underwent T4-L2 posterior spinal fusion surgery with concave RR and convex DRC using the outrigger. After the apical setscrews were tightened (T7–11), segmental DVR for thoracolumbar curve-correction was performed from T11 to the lowest instrumented vertebra (L2). After surgery, TK increased to 28°. Next, a thirteen-year-old girl with Lenke 1B- was shown (Figure 5c,d). A preoperative X-ray showed thoracic hypokyphosis of 2°. She was found to have six non-rib-bearing vertebrae (L1–6). Apical five vertebral kyphosis was 0 degrees. (b) She underwent T5-L2 posterior spinal fusion surgery with concave RR and convex DRC using the outrigger. After the apical setscrews were tightened (T6–10), segmental DVR for thoracolumbar curve correction was performed from the caudal neutral vertebra (T12) to the lowest instrumented vertebra (L2). After surgery, TK was increased to 28°.

## 4. Discussion

The concave RR with an outrigger group showed a significantly greater TK and a better correction rate than those of the RR in the no outrigger group. Apical five vertebral kyphosis was also significantly more substantially kyphotic in the outrigger group. At the same time, changes in SPi and RHi in the outrigger group were significantly better than those in the without outrigger group.

**Figure 6:**
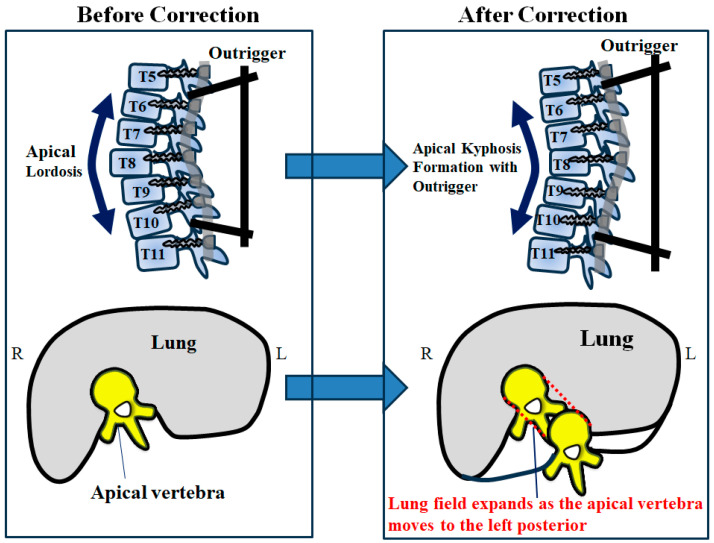
Schema of lung field expansion due to apical kyphosis correction with outriggers.

A schema of the results is shown in Figure 6. Based on substantial apical kyphosis correction, the apical vertebra moved to the posterior in the sagittal plane and, at the same time, shifted toward the midline due to scoliosis correction in the coronal plane. Therefore, it was thought that lung fields expanded in the cross-sectional CT analysis because the apical vertebra moved to the left posterior in the axial plane following surgery with an outrigger, as shown in Figure 6. These facts suggest that the substantial kyphosis formation of the periapical vertebrae by the outrigger resulted in an expansion of the lung field and an improvement in RHi.

Our previous studies have shown that concave rods flattened during concave RR in posterior spinal fusion. We have been trying to correct apical kyphosis by the compensatory overbending of the concave rod. However, we have noticed that the rod loses stiffness with compensatory overbending, making it easier to flatten. Previous reports that notch formation due to rod bending, bending back, and repeated bending with a French bender cause a decrease in rod stiffness and yield strength [10,11,28]. Therefore, we thought that the more times the concave RR is bent, the less stiff it becomes. If we use a stiffer and stronger rod, such as 6.0 mm CoCr, it may cause screw-loosening and subcutaneous protrusion due to its bulk, since many girls with AIS are relatively thin. For the above reasons, we devised a surgical method using an outrigger to temporarily increase the stiffness of the 5.5 mm CoCr rod during concave RR and to maintain a corrective force to the spinal column prior to removal of the outriggers. It was considered that the use of the outrigger device would reduce complications such as subcutaneous protrusion or screw-loosening during surgery because the implant with a small rod diameter could be used. In fact, when we used an outrigger device with a 5.5 mm CoCr rod, we did not find any cases where the screw loosened or backed out during concave RR.

Regarding the safety of neurological problems, there was a case in which signal changes during spinal cord monitoring occurred in the group without an outrigger, whereas no signal changes were observed in the RR with outrigger group. Signal changes in spinal cord monitoring during surgery are most frequently associated with spinal traction or patient positioning [29]. Additionally, animal models have demonstrated that the surgical distraction of the spinal canal results in histological evidence of spinal cord injury [30,31,32]. We also reported that spinal canal elongation after surgery in AIS patients with Lenke type 1 or 2 was observed in a multicenter study [33]. These facts suggest that concave RR and convex DRC with an outrigger might prevent excessive spinal canal elongation during surgery in patients with AIS.

We analyzed the concave rod’s deformation during RR, as shown in Figure 1. The concave rod deformed by approximately 15° during RR due to the corrective force of the scoliotic spine [6]. The reduction force [34] and fatigue life [35] of the CoCr rod were stronger than those of titanium. The correctional force produced by the titanium 30° pre-bended rod was approximately 67% that of CoCr [34]. However, if the CoCr rod was plastically deformed after a reduction in rigid spinal deformity, the force of the CoCr rod in maintaining its original shape was lower than that of titanium, stainless steel, and ultrahigh-strength stainless steel [34]. This phenomenon suggests that it is important to prevent the plastic deformation of the concave CoCr rod. That is, the outrigger device may be useful for preventing the plastic deformation of the concave rod because the outrigger device that is removed after dual CoCr rods contains the main thoracic curvature. Our results also suggest that there were significant differences when obtaining the TK angles and apical five vertebral kyphosis between the groups using RR with and without an outrigger. These phenomena suggest that dual CoCr rods may continuously contribute to maintaining the contour of bended rods due to their stiffness. If titanium rods were used for correction, it is presumed that the results would be different. As far as we know, there have been no reports showing the effectiveness of outrigger devices that suppress plastic deformation during concave rod RR. However, if the scoliotic curvature was severely rigid, dual CoCr rods might be flattened after the outrigger device was removed. We cannot evaluate how much correction loss occurred after the outrigger device was removed because the outrigger device was not permanently attached. Therefore, further studies will be needed in the future.

We performed segmental uniplanar pedicle screw fixation with concave RR without an outrigger, followed by DRC and segmental DVR, for thoracic and thoracolumbar curvature in Lenke type 1 or 2 patients with AIS. We performed an analysis of how much concave RR, DRC, and segmental DVR in the thoracic spine contribute to the derotation of the apical vertebral bodies using an intraoperative cone-beam CT [6]. The concave RR had little effect on apical vertebral body derotation, and convex DRC was reported to be effective in improving vertebral body rotation by approximately 6° [6]. Segmental DVR for the apical thoracic curve additionally improved vertebral body rotation by approximately 3° in our study [6]. When correcting the AVR angle, there was no significant association between the degree of correction and the use or nonuse of an outrigger. In our results, the AVR was corrected by about 8° in the RR without the outrigger, and by 7.9° with the outrigger. These findings suggest that convex DRC is the more effective technique for apical vertebral derotation, compared with segmental DVR. The technique of using concave RR and DRC with the outrigger was not performed for segmental DVR in apical thoracic curvature. If segmental DVR was performed for apical thoracic curvature, the corrected apical hypokyphosis might be lost. These results suggest that segmental DVR for an apical thoracic spine in concave RR with the outrigger does not affect the deterioration of AVR.

Significant improvements were noted in TK and apical five vertebral kyphosis both with and without the outrigger (Figure 3). In the scoliotic bone model study, it was clear that the use of concave RR with the outrigger was able to maintain the TK. If TK was obtained in patients with AIS, LL might increase, since sagittal alignment of the spine was kept harmonious, following strict rules, under physiological conditions [36]. Although our data did not show an increasing LL, this might change after long-term follow-up. On the other hand, RHi and SPi significantly improved in concave RR that used the outrigger compared to the concave RR performed without the outrigger. Since RHi and SPi are measured in the axial plane by CT, there is a possibility that lung volume and the rib hump will be improved when the apical spine is moved posteriorly. Increasing TK in concave RR with the outrigger suggests that the apical vertebra of thoracic scoliosis might move posteriorly. RHi and SPi were significantly improved after concave RR surgery with the outrigger. In fact, it has been reported that TK correlates with SPi, and RHi correlates with the main thoracic Cobb angle in patients with AIS [37]. Our data showed a 6° difference between the two groups after surgery, although the difference may be difficult to prove to be clinically superior. However, obtaining the TK angle was thought to contribute to improvements in RHi and SPi after surgery. These factors, which directly led to improvements in the rib hump and vital capacity, were most important in patients with AIS.

There are several limitations in this study. First, the patients in the two groups were not randomized and the numbers of participants in each group differed. We devised the outrigger device and studied a surgical method that could temporarily increase the strength of the concave rod during RR. Since the patients were selected for their willingness to be followed for >2 year after the surgical method was established, the number of cases and follow-up periods between the two groups varied. We plan to increase the number of cases and re-evaluate these findings in the future. Second, if the outrigger device was not firmly attached, loosening between the outrigger device and the concave rod could result in the poor correction of apical hypokyphosis. This is also an important point in the surgical methodology and could be prevented if it was confirmed that the outriggers could be firmly fixed in parallel to the concave rod. However, this outrigger system has a support bar that can be firmly fixed during concave RR and its rotations are designed to be as parallel as possible. Therefore, we used this support bar during surgery to avoid this problem. Finally, it was not possible to assess how much correction loss occurred after removing the outriggers. In the case of rigid scoliotic curvature, if the outriggers cannot be supported by two 5.5 mm CoCr rods when the outriggers are removed, some correction loss might occur. Further research is needed.

## 5. Conclusions

The surgical technique using concave RR and convex DRC with an outrigger device to maintain the concave rod’s contour in Lenke type 1 or 2 patients with AIS is advantageous for correcting apical hypokyphosis, followed by the subsequent formation of TK. Improvements in RHi and the expansion of the pulmonary area were observed on a cross-sectional CT with the use of outriggers.

## Figures and Tables

**Table 1:** Basic characteristics of patients with AIS.

	RR without Outrigger (*n* = 41)	RR with Outrigger (*n* = 36)	*p*-Value *
Age (y)	14.1 ± 2.0	14.0 ± 1.5	0.81
Height (cm)	155.0 ± 6.4	154.9 ± 8.3	0.68
Weight (kg)	46.0 ± 6.3	45.7 ± 6.3	0.51
Risser (grade)	3.4 ± 1.1	3.0 ± 1.5	0.14
Preoperative radiographic factors			
PT (°)	24.9 ± 10.9	22.7 ± 11.5	0.35
MT (°)	52.2 ± 7.8	52.7 ± 9.8	0.76
TL/L (°)	19.9 ± 6.1	18.9 ± 4.8	0.52
TK (°)	18.2 ± 8.9	17.1 ± 8.4	0.25
LL (°)	45.0 ± 12.5	42.5 ± 9.2	0.19
AVR (°)	15.1 ± 7.0	14.5 ± 6.9	0.37
TAVT (mm)	39.6 ± 15.6	38.6 ± 14.7	0.88

RR; rod rotation, PT; proximal thoracic, MT; main thoracic, TL/L; thoracolumbar/lumbar, TK; thoracic kyphosis, LL; lumbar lordosis, AVR; apical vertebral rotation, TAVT; thoracic apical vertebral translation. * Statistical analysis was performed to compare the RR without an outrigger with the RR with an outrigger using an unpaired *t*-test.

**Table 2:** Surgical outcomes of concave RR with and without outrigger.

	Rod Rotation without Outrigger (*n* = 41)	Rod Rotation with Outrigger (*n* = 36)	*p*
Postoperative radiographic factors			
PT (°)	11.1 ± 4.1	10.6 ± 3.9	0.68
MT (°)	14.5 ± 3.6	12.6 ± 3.6	0.21
TL/L (°)	9.1 ± 3.2	10.0 ± 2.7	0.42
TK (°)	21.8 ± 7.2	27.0 ± 5.9	0.002 *
LL (°)	45.0 ± 12.5	49.1 ± 10.5	0.08
Total number of fixed vertebrae	10.0 ± 1.8	9.8 ± 1.5	0.78
Correction rate (%)	72.1 ± 6.4	75.6 ± 5.5	0.03 *
Correction angle of AVR (°)	8.0 ± 6.1	7.9 ± 4.2	0.20
Correction rate of TAVT (%)	82.6 ± 22.8	84.3 ± 14.1	0.24

RR; rod rotation, PT; proximal thoracic, MT; main thoracic, TL/L; thoracolumbar/lumbar, TK; thoracic kyphosis, LL; lumbar lordosis, AVR; apical vertebral rotation, TAVT; thoracic apical vertebral translation. * Statistical analysis using unpaired *t*-tests was performed to compare the RR without outrigger to the RR with outrigger groups’ results.

## Data Availability

The data presented in this study are available on request from the corresponding author.
